# Genome analysis of clinical multilocus sequence Type 11 *Klebsiella pneumoniae* from China

**DOI:** 10.1099/mgen.0.000149

**Published:** 2018-02-08

**Authors:** Ning Dong, Rong Zhang, Lizhang Liu, Ruichao Li, Dachuan Lin, Edward Wai-Chi Chan, Sheng Chen

**Affiliations:** ^1^​ Shenzhen Key lab for Food Biological Safety Control, Food Safety and Technology Research Center, Hong Kong PolyU Shen Zhen Research Institute, Shenzhen, PR China; ^2^​ State Key Lab of Chirosciences, Department of Applied Biology and Chemical Technology, The Hong Kong Polytechnic University, Hung Hom,Kowloon, Hong Kong SAR; ^3^​ Second Affiliated Hospital of Zhejiang University, hospital of Zhejiang University, Hangzhou, PR China

**Keywords:** *Klebsiella pneumonia*, ST11, ST258, genome analysis, cps loci, virulence, antimicrobial resistance

## Abstract

The increasing prevalence of KPC-producing *Klebsiella pneumoniae* strains in clinical settings has been largely attributed to dissemination of organisms of specific multilocus sequence types, such as ST258 and ST11. Compared with the ST258 clone, which is prevalent in North America and Europe, ST11 is common in China but information regarding its genetic features remains scarce. In this study, we performed detailed genetic characterization of ST11 *K. pneumoniae* strains by analyzing whole-genome sequences of 58 clinical strains collected from diverse geographic locations in China. The ST11 genomes were found to be highly heterogeneous and clustered into at least three major lineages based on the patterns of single-nucleotide polymorphisms. Exhibiting five different capsular types, these ST11 strains were found to harbor multiple resistance and virulence determinants such as the *bla*_KPC-2_ gene, which encodes carbapenemase, and the yersiniabactin-associated virulence genes irp, ybt and fyu. Moreover, genes encoding the virulence factor aerobactin and the regulator of the mucoid phenotype (*rmpA*) were detectable in six genomes, whereas genes encoding salmochelin were found in three genomes. In conclusion, our data indicated that carriage of a wide range of resistance and virulence genes constitutes the underlying basis of the high level of prevalence of ST11 in clinical settings. Such findings provide insight into the development of novel strategies for prevention, diagnosis and treatment of *K. pneumoniae* infections.

## Data Summary

1. All Genomes sequenced in this study have been deposited in GenBank with Bioproject numbers PRJNA422332 and PRJNA422171.

2. The information for the reference strains selected from GenBank is listed in supplementary dataset S1 (available in the online version of this article).

3. All information on SNPs has been included in supplementary dataset S2 and uploaded in the mGen dropbox.

4. Information on plasmid replicon genes and antimicrobial resistance genes harbored by these strains is included in supplementary dataset S3.

5. Information on MLST and virulence genes harbored by these strains is included in supplementary dataset S4.

6. Data in the mGen dropbox can be assessed through: https://secure-web.cisco.com/1GOFegc745a2-v6M3WPEtvlvvKOQ9r096PvgnLQGJDDKRbXDUy-lQ7gL6BziV79yYdQWOV_ht-ZhVdMtorIbRtrYRYjk9uurSDAYpTdpF34zgvZtlyJZOehkQiKyUKCjovlp1SJA9VrnxvfzhXGM-mEZsUAZwTy7Lvn9jk1O3OfV6dvmaZNHQYSXBu6xWXy7Qh2CFw5attKJtkciEZ-qAYLJOFV1WXPA5P1_2chAwWtkzFMvK82xsZZzLFUEYjvd8oXdaa3b14oHEcXYMj7DBMG0nHqSnXn9aoiv3ORMG4TwbPahZDlkp8WtHY-cWRt_B/https%3A%2F%2Ffigshare.com%2Fs%2Fbb6d3649a9f6efdbaa8f

**Impact Statement**

A comprehensive genomic analysis of 58 clinical strains of ST11 *K. pneumoniae*, the dominant KPC-producing clinical clone in China, was performed in this study. We found that that these strains, collected from geographically diverse locations in the country, were genetically diverse and could be segregated into three clades, each of which exhibited distinct capsule polysaccharide (*cps*) loci. Various antimicrobial resistance genes and virulence genes, such as those encoding salmochelin, aerobactin and RmpA, the hallmarks of hypervirulent *K. pneumoniae,* were detected in the genomes of these strains. This study provides insights for the development of strategies for prevention, diagnosis and treatment of ST11 clinical infections.

## Introduction

*Klebsiella pneumoniae* has gained notoriety as a major opportunistic pathogen which causes a range of hospital-acquired infections [1]. The emergence of multidrug-resistant *K. pneumoniae* strains, which cause untreatable infections, has resulted in extensive public concern [2]. Notably, *K. pneumoniae* carbapenemase (KPC)-producing *K. pneumoniae*, one of the most clinically significant carbapenem-resistant Enterobacteriaceae (CRE) strains, has not only been disseminated globally, but is also associated with high morbidity and mortality rates [3]. To date, 16 different KPC variants (KPC-2–KPC-17), classified on the basis of single-amino-acid mutations, have been identified, among which KPC-2 and KPC-3 are the best-studied enzymes [2, 4]. Transmission of the *bla*_KPC_ gene involves multiple mechanisms ranging from clonal spread to horizontal transfer mediated by plasmids and other transposable genetic elements, notably Tn*4401* [5].

The clinical prevalence of KPC-producing *K. pneumoniae* has been largely attributed to dissemination of strains of the clonal group (CG) 258, with ST258 and ST11 being the dominant multilocus sequence types (ST) [3, 5–8]. ST258 has disseminated worldwide since its emergence during the early to mid-2000s, especially in North America, Latin America and several countries in Europe [5, 8, 9]. In Asia and South America, however, the dominant KPC-producing clone is ST11 [8, 10], which is a single locus (*tonB*) variant of ST258, with the *tonB4* gene in ST11 differing from *tonB79* in ST258 by four single-nucleotide polymorphisms (SNPs) [10]. According to Gaiarsa *et al.*, a putative recombination event (which happened sometime before 1985), during which a donor related to *K. pneumoniae* ST1628 contributed approximately 1.3 Mbp to an ancestor of ST11 (CG258), gave rise to CG258 [11]. Results from a recent genome-based study indicated that ST258 *K. pneumoniae* differed significantly from ST11 in terms of genetic composition. ST258 was found to be a hybrid clone, with 80 % of its genome derived from ST11-like strains and 20 % from ST442-like strains [2, 5, 12]. Strains of the ST258 type were found to comprise at least two distinct lineages, namely clade 1 and clade 2, which differ mainly in a genomic region where the gene encoding capsule polysaccharide (CPS), *cps*, is located [6]. In contrast, the genetic composition of ST11, which is the dominant carbapneme-resistant *K. pneumoniae* clone in China, remains poorly understood [10, 13]. The complete genomic sequences of two clinical ST11 multidrug-resistant strains, HS11286 and JM45, are publicly available and widely used as references for various genetic studies [5, 14–16]. A previous study demonstrated that the ST11 genomes exhibited a relatively high degree of diversity [17]. Jiang *et al.* tracked the outbreak of ST11 *K. pneumoniae* in a single Chinese hospital by performing whole-genome sequencing of 12 strains; in this previous work, phylogenetic analysis resulted in partitioning of these strains into three separate clades [16]. However, a comprehensive clonal lineage map of ST11 strains prevailing in China is currently not available. Also, data regarding the pathogenicity and antimicrobial resistance profiles of the ST11 strains is scarce. To address these issues, we performed whole-genome sequencing to obtain the complete genome of one KPC-producing ST11 clinical isolate, followed by phylogenetic analysis and genome mining of a total of 58 ST-11 clinical isolates collected from diverse geographic locations in China. Findings from this work led to the identification of unique genetic traits of the ST11 clone and helped promote better understanding of the genetic basis of virulence of this important clone.

## Methods

### *K. pneumoniae* isolates and sequencing

Thirty-one carbapenem-resistant *K. pneumoniae* isolates were collected from blood, sputum and stool specimens of patients at eight hospitals located in different provinces of China. The isolates were non-outbreak-related and randomly selected from various locations in China. Antimicrobial susceptibilities were determined by the agar dilution method according to the Clinical and Laboratory Standards Institute (CLSI) guidelines [18]. Genomic DNA was extracted from overnight cultures by using the PureLink Genomic DNA Mini Kit (Invitrogen). Genomic libraries were prepared with an approximately 350 bp insert size using the NEBNext Ultra DNA Library Prep Kit (New England Biolabs) and sequenced with the Illumina NextSeq 500 sequencing platform. One of these strains, GD4, was simultaneously selected for sequencing with the PacBio RSII single-molecule real-time (SMRT) sequencing platform (Wuhan Institute of Biotechnology). All ST-11 *K. pneumoniae* genomes that were publicly available in the NCBI Pathogen Detection database (https://www.ncbi.nlm.nih.gov/pathogens/) as of January 2017 (a total of 27 genomes), were also included for analysis in this study. Genetic information for the test isolates is listed in File S1.

### Genome assembly and annotation

Raw reads generated in this study and the Illumina reads of 11 strains obtained from the NCBI database were trimmed or filtered to remove low-quality sequences and adaptors. Both Illumina and PacBio reads were *de novo* assembled with the SPAdes Genome Assembler v3.9.1 [19, 20]. Illumina reads of strain GD4 was aligned to the corresponding PacBio contigs to improve the accuracy of the genome sequence data and obtain the complete genome sequence of strain GD4. The 30 draft and 1 complete genome sequences generated in this study and the 27 genome sequences retrieved from the NCBI database were all annotated with the RAST tool [21] and Prokka [22].

### Genome profiling

Genome profiling was conducted using the assembled genome sequences. Integrative and conjugative elements (ICEs) were predicted as described previously [15]. Acquired antibiotic resistance genes were identified with ResFinder 2.1 (https://cge.cbs.dtu.dk/services/ResFinder/) [23]. Plasmid replicons were analyzed using PlasmidFinder (https://cge.cbs.dtu.dk/services/PlasmidFinder/) [24]. Insertion sequences (ISs) were identified using Isfinder and ISsaga (https://www-is.biotoul.fr/index.php) [25]. Phage-associated regions were identified by PHAST (http://phast.wishartlab.com/index.html) [26]. Multilocus sequence types, virulence-associated genes encoding yersiniabactin, aerobactin and salmochelin and the regulators of the mucoid phenotype were determined with Kleborate [27]. The heatmaps of the antimicrobial resistance genes and virulence determinants were generated using an in-house script.

### Phylogenetic analysis

The harvest suite, which was designated for analyzing intraspecific microbial genomes and encompasses three modules (Parsnp, Gingr and HarvestTools), was applied to filter recombination (-x), run core genome alignment and variant calling and reconstruct the phylogenetic tree with the 58 assembled genomes using default settings [28]. Interactive tree of life (iTOL) v3 (http://itol.embl.de/) was applied to modify and visualize the reconstructed phylogenetic tree [29]. Genome assemblies for two representative ST258 *K. pneumoniae* isolates, NJST258_1 (GenBank accession number CP006923) and NJST258_2 (GenBank accession number NZ_CP006918), and one CG258-unrelated isolate, *K. pneumoniae* MGH78578 (ST38, GenBank accession number AB720665) were retrieved from GenBank and used to reconstruct a phylogenetic tree with three ST11 isolates (each from a different evolutionary clade) using the harvest suite. This tree, as well as the variants, was visualized with gingr [28]. The chromosome sequence of *K. pneumoniae* strain GD4 was used as a reference for both phylogenetic analyses.

### Core genome and pan-genome analysis

Conserved core genes among the ST11 isolates were analyzed with Roary [30] with a blastp percentage identity of 95 %. The Prokka [22] annotated files were used as inputs for Roary. The parameter for the percentage of isolates a gene must be in to be classified as core was set to be 99 %.

### Comparative genomics analysis

Genome sequences of *K. pneumoniae* ST11 strains GD4, JM45 and HS11286, originating from China, were compared with seven other closed genomes, ATCC BAA-2146, NJST258_1, NJST258_2, NTUH-K2044, MGH78578, CG43 and KCTC2242, using the blast Ring Image Generator (BRIG) [31]. Capsular typing was performed using Kaptive [32]. Comparison of the *cps* locus was conducted by using EasyFig [33]. Plasmid homology search was performed for the plasmid pKPGD4 harbored by strain GD4 using blastn on NCBI's nucleotide collection (nr/nt) database. Sequence comparisons between plasmid pKPGD4 and related plasmids were also conducted with BRIG [31].

## Results and discussion

### Genome sequence of carbapenem-resistant ST11 *K. pneumoniae* clinical isolates

**Fig. 1.**
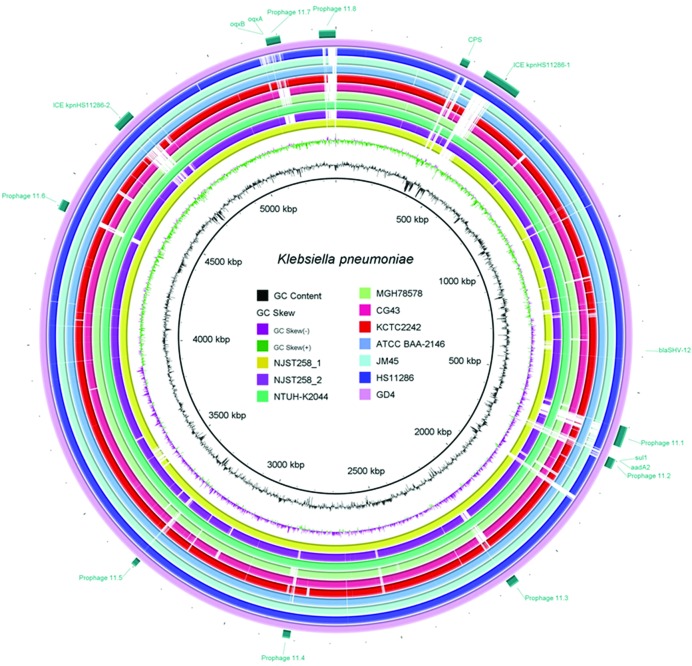
Alignment of the *K. pneumoniae* genomes. A total of ten *K. pneumoniae* genomes were compared using the chromosome sequence of strain GD4 (outermost circle) as a reference. Prophages (prophages 11.1–11.8), integrated conjugative elements (ICE*Kpn*HS11286-1 and ICE*Kpn*HS11286-2) and the capsule polysaccharide region are indicated by rectangles. Antimicrobial resistance genes are indicated.

ST11 was found to be the dominant sequence type of carbapenem-resistant *K. pneumoniae* strains in China, accounting for 60 % of such strains [34]. *K. pneumoniae* has been reported to be highly genetically diverse, with a large accessory genome that comprises 30 000 protein-coding genes [35]. In this study, the complete genome sequence of a multidrug-resistant *K. pneumoniae* strain GD4 isolated from a sputum specimen in 2015 at Huashan Hospital, Guangdong, PR China (MIC profile in File S1) was obtained. The genome of this strain was found to comprise a chromosome of 5 366 808 bp in size, and a 170 821 bp plasmid designated pKPGD4. The overall DNA G+C content of the chromosome, in which 5215 coding sequences (CDSs) and 80 tRNA molecules were detectable, was 57.5 %. The genetic diversity of *K. pneumoniae* genomes has been previously demonstrated to be primarily due to elements that migrate frequently by horizontal gene transfer, including plasmids, phages, integrated conjugative elements (ICEs) and insertion sequences (ISs) [36]. Resembling the ST258 strains [6], the chromosome of strain GD4 was found to harbor numerous mobile genetic elements including eight putative prophages (designated prophages 11.1–11.8), two ICEs (designated ICE*Kpn*HS11286-1 and ICE*Kpn*HS11286-2 based on the name of the parent strain HS11286) and 40 insertion sequences (ISs) (Fig. 1). The chromosomal features of three completely sequenced ST11 *K. pneumoniae* strains, namely GD4, HS11286 and JM45, were compared and are summarized in Table 1. Information on all 58 strains tested in this study is listed in File S1. Apart from the completely assembled sequences, the genomes of other ST11 isolates in this study were found to comprise 74–161 scaffolds. Like other *K. pneumoniae* genomes [6], the genome sizes of the ST11 strains ranged from approximately 5.4 Mbp to around 5.7 Mbp. Pan-genome analysis identified a total of 8285 genes in the genomes of the 58 ST11 *K. pneumoniae* isolates, among which 4297 (about 82.4 % of the total genes in isolate GD4) were core to all isolates. The ST11 *K. pneumoniae* clone harbors a large set of accessory genes to render the clone adaptable to different environments.

**Table 1.** Comparison of key features of the ST11 chromosomes

	GD4	HS11286*	JM45†
Size (bp)	5 366 808	5 332 752	5 273 813
DNA G+C content (%)	57.5	57.5	57.5
Number of CDS	5215	5316	4872
rRNA	25	25	25
16S	8	8	8
23S	8	8	8
5S	9	9	9
tRNA number	80	87	83
tmRNA number	1	1	1
Plasmid number	1	6	2
Prophages	8	7	8
ICEs number	2	2	1
ICE*Kpn*HS11286-1	+	+	−
ICE*Kpn*HS11286-2	+	+	+
IS number	40	30	24
ISNCY	7	6	7
IS6	2	−	−
IS5	15	8	−
IS481	1	−	1
IS3	10	8	15
IS1380	1	6	1
IS1182	1	−	−
IS1	3	1	−
IS66	−	1	−
*bla* genes	*bla*_KPC-2_, *bla*_CTX-M-65_, *bla*_TEM-1_	*bla*_KPC-2_, *bla*_CTX-M-14_, *bla*_TEM-1_	*bla*_KPC-2_, *bla*_CTX-M-24_, *bla*_VEB-3_

ICE, integrative and conjugative elements; IS, insertion sequence.

*Data for this column are from reference [15].

†Data for this column are from reference [12] except for IS numbers which are derived from this study.

### Phylogenetic analysis

**Fig. 2.**
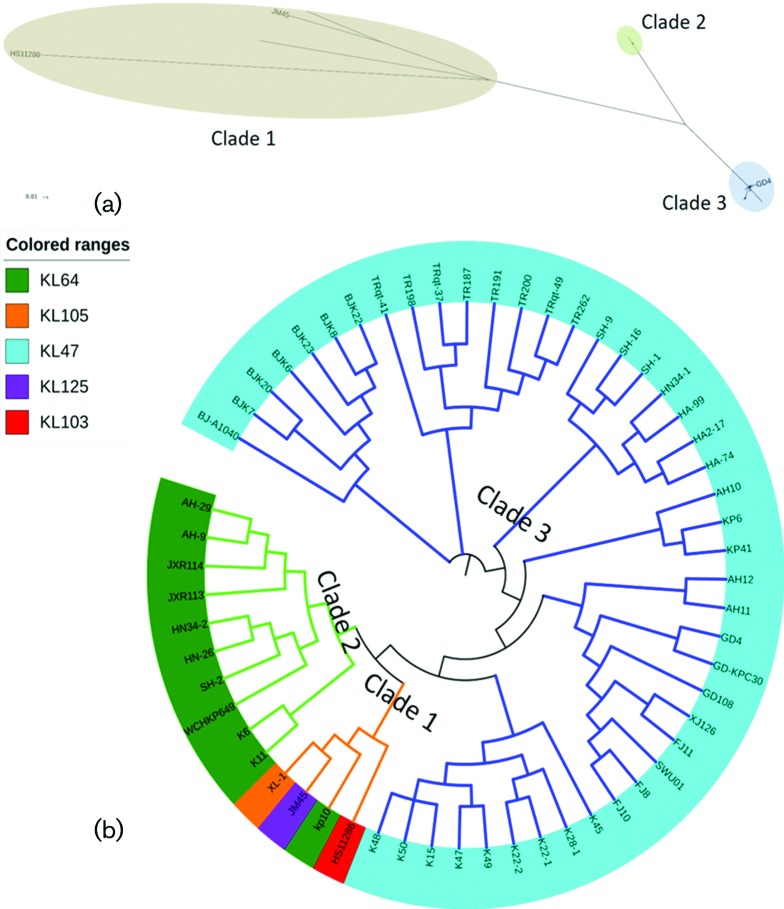
Approximately maximum-likelihood phylogeny estimated with the parsnp software [28] based on a total of 6749 unique concatenated SNPs in the core genome of 58 clinical ST11 *K. pneumoniae* strains from China. (a) Unrooted phylogenetic analysis of 58 *K. pneumoniae* clinical isolates. GD4, HS11286 and JM45 are ST11 strains with completed genome sequences. Bar, 0.01 substitutions per nucleotide position. (b) Circular phylogenetic tree of the 58 strains. The lengths of the branches are not proportional to the evolutionary distances in the cladogram. Different background colors underneath each strain name indicate the K locus type of the corresponding strain detected by Kaptive [32].

After filtering the recombination, a 4 205 312 bp conserved core genome was identified among the genomes of the 58 ST11 strains. A total of 6749 SNPs were identified in the core genomes and were used to reconstruct an approximately maximum-likelihood phylogenetic tree (File S2). Similar to the ST258 *K. pneumoniae* strains, which comprised at least two distinct lineages [6], the 58 ST11 isolates were found to be clustered into three major groups, namely clade 1, clade 2 and clade 3 with respectively 4, 10 and 44 strains in each clade (Fig. 2). Isolates within clade 1 and clade 2 were found to differ from those of clade 3 by an average of 2848 (a range of 2595–3353, around one SNP per 1884 nucleotides) and 1198 (a range of 1181–1244, about one SNP per 4480 nucleotides) SNPs, respectively (File S2). This finding was similar to those of a previous study, in which the 12 ST11 outbreak strains from a single hospital could also be grouped into three clades [16], indicating that the ST11 lineage should not be regarded as a single clone. It should be noted that strains in clades 2 and 3 of the previous study were mostly clustered in clade 3 of this study, whereas, strains in clade 1 of the previous study was clustered into clade 2 in this work (Fig. 2). Importantly, none of strains belonging to clade 1 in this study was detectable in the previous study. The discrepancy between the clustering data from the two studies was probably due to the large sample size and inclusion of more genetically diverse strains in our work. Phylogeographic clustering could not be properly resolved in the current study, owing to the small number of strains and presence of different lineages at a single location.

**Fig. 3.**
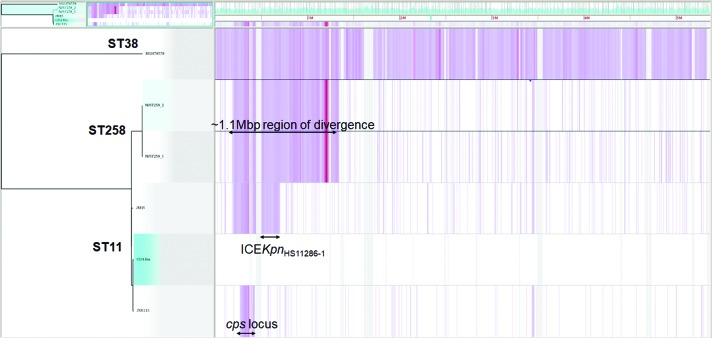
Phylogenetic tree and SNPs of representative *K. pneumoniae* strains from CG258. Pink lines indicate SNPs identified with the harvest suite [28]. Regions of divergence were labelled with double side arrows.

Using ST38 *K. pneumoniae* isolate MGH78578 as an outgroup, the CG258 isolates could be grouped into a two-branched clade which shows extensive variations compared with the ST38 clade (Fig. 3). The ST258 lineage harbors an approximately 1.1 Mbp region divergent from the ST11 lineage which have been proposed to have originated from an ST442-like clone [12]. This finding is consistent with the results of a previous study, which indicated that members of CG258 descend from a common ancestor (mostly of ST11) and then diversified into distinct lineages [17]. The ST11 lineage, which was grouped into three clades in this study, were mainly classified by the *cps* locus it harbors as displayed in the variation regions in Fig. 3.

### Comparison of the *cps* cluster

The genomes of ST258 and ST11 *K. pneumoniae* strains have been reported to differ mainly in the composition of an approximately 1.1 Mbp region in which the *cps* locus is located [12]. SNPs analysis showed that the most diversified area in this region was the *cps* operon, which encoded the capsule polysaccharide (CPS), a known key virulence determinant in *K. pneumoniae* [2]. Large-scale recombination events and capsule switches are known to contribute to the strain variation within CG258 [17]. Traditional serological typing has been used to identify more than 78 distinct capsular types (K types) in *K. pneumoniae* [2, 6, 17, 37]. Recently a novel program (Kaptive) for identifying the CPS synthesis loci (K loci) has been developed by using the full locus information extracted from the genome data, the use of which resulted in an increasing in the total number of known K loci to 134 [32]. By adopting the Kaptive program in our study, the K types of 58 ST11 isolates were identified (Fig. 2b). In line with the previous finding that two distinct *cps* gene clusters were involved in segregating the ST258 clinical isolates into two distinct clades [17], the *cps* loci of the ST11 strains could also be segregated into phylogenetically defined sublineages. The 10 strains in clade 2 were KL64 and all the 44 strains in clade 3 were found to belong to KL47. On the other hand, the four strains (HS11286, JM45, kp10 and XL-1) within clade 1 were found to belong to different K types, KL103, KL125, KL64 and KL105. This is not surprising as the four strains did not exhibit significant genetic relatedness, even though we manually grouped them into one cluster (Fig. 2a). On the basis of these observations, we speculated that the ST11 ‘strain’ has undergone a number of capsular exchanges since its emergence as a major nosocomial outbreak agent. Notably, the serotype of strain HS11286 has been previously characterized as K74 through *wzi* typing [17]. However, the 78 different *wzi* loci could be clustered into four homology groups, indicating that *wzi* typing has lower discrimination power compared with *cps* loci typing [37]. By using the complete locus sequence, it is reasonably to redefine HS11286 as the novel type, KL103 based on the genetic sequence of its *cps* locus. Some novel capsular types have been reported to be predominant among clinical carbapenem-resistant *K. pneumoniae* strains in Italy and the USA [6, 38, 39].

**Fig. 4.**
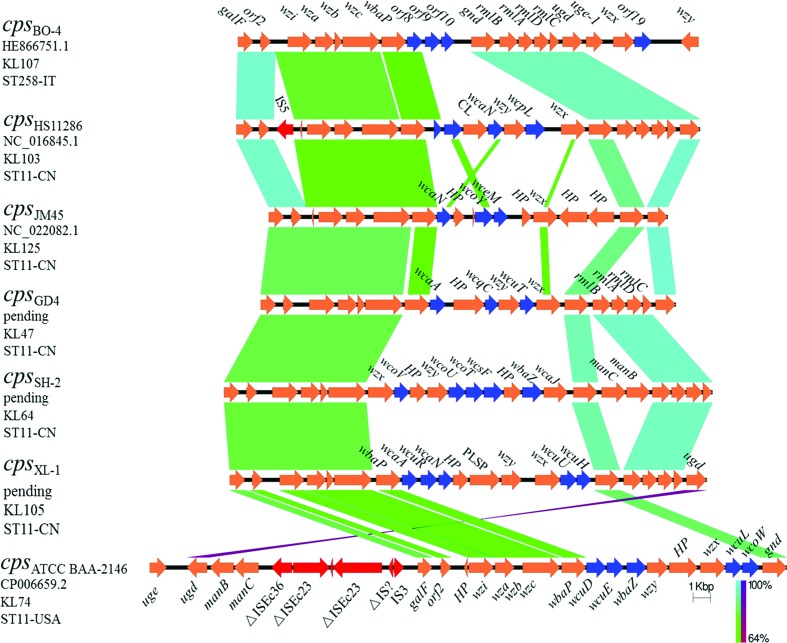
Comparison of the *cps* gene clusters from *K. pneumoniae* strains of CG258. The order of the pairwise comparisons is defined by the phylogenetic relationships. Locus types KL47 and KL64 correspond to serotypes K47 and K64, respectively. K types KL103, KL125 and KL105 are locus types which have not been phenotypically defined and are defined from DNA sequence data on the basis of gene content [32]. Sequence origins, accession numbers and STs for the respective strains are indicated. Arrows indicate the direction, relative length and function of ORFs. The *cps* loci of *K. pneumoniae* strains ATCC BAA-2146 and BO4 were downloaded from the NCBI database and used as references for the comparison. ORFs encoding transposases are colored in red, while those encoding non-initial glycosyltransferases are colored in blue. HP, CL and PLSP are short for hypothetical protein, carbohydrate lyase and pectate lyase superfamily protein, respectively. Homologous regions are connected by areas of different colors reflecting the degree of nucleotide identity (from 64 to 100 %).

The common genetic features of the *cps* clusters in *K. pneumoniae* strains have been determined previously [2]. Generally, the *cps* locus harbored a highly conserved 5′ end region of six genes (*galF*, *orf2*, *wzi*, *wza*, *wzb* and *wzc*) whose products are responsible for CPS assembly and translocation with sequence variations often observable in the central and 3′ end regions (from *gnd* to *ugd*) [37, 40]. The structures of the five distinct loci detected in this study as well as two reference loci types (*cps*_BO-4_ and *cps*_ATCC BAA-2146_) reported in previous studies [41, 42] were compared and are shown in Fig. 4 and Table S2. Briefly, the *cps* loci of the Chinese ST11 strains ranged from approximately 23 kpb to approximately 28 kbp with 18–23 ORFs (Table 2). Synthesis of the capsular repeat in *K. pneumoniae* is mediated by the initial glycosyltransferase (GT) WbaP or WcaJ, which catalyze transferal of galactose-1-phosphate or glucose-1-phosphate to undecaprenol phosphate, respectively [37]. The *wcaJ* gene 5was found only in the clade 2 strains and one strain (kp10) in clade 1, and the initial GTs genes for other ST11 strains were all found to be *wbaP*, indicating that different saccharides might be present in the repeat unit of the capsule. Addition of sugars in the capsule is catalyzed by specific non-initial GTs [43]. In the case of ST11 *K. pneumoniae*, strains within distinct clades were found to harbor different combinations of non-initial GT genes (Fig. 4, blue arrows) in the central *cps* region. For instance, clade 3 strains such as GD4 were found to harbor *wcaA*, *wcqC* and *wcuT* genes in the central region, whereas clade 2 strains such as SH-2 contained *wcoV*, *wcoU*, *wcoT*, *wcsF* and *wbaZ*. The 3′ end *gnd–ugd* region of the *cps* locus which harbored the gene clusters *manCB* or *rmlBADC*, which are known to be responsible for the synthesis of GDP-d-mannose or dTDP-l-rhamnose in the majority of capsular types [37]. In this work, the finding that the *cps* types KL107 (*cps*_BO-4_), KL103 (*cps*_HS11286_), KL47 (*cps*_GD4_) and KL105 (*cps*_XL-1_) harbored the *rmlBADC* gene cluster, is highly consistent with the phenomenon of the presence of rhamnose in the repeat units of the capsule [37]. On the other hand, the presence of *manCB* genes does not always correlate with the existence of mannose in the capsule [37]. Both *manCB* and *rmlBADC* gene clusters were detected in the KL64 *cps* type (*cps*_SH-2_) and neither of the two clusters was found in the KL125 type (*cps*_HS11286_). Additionally, genes for capsule modification was detected in the *cps* loci of some strains, such as those encoding carbohydrate lyase in HS11286 and the pectate lyase superfamily protein-encoding gene in XL-1 (Fig. 4), indicating that distinct modifying systems were responsible for production of different capsule structures. It should be noted that when T11 and ST258 were compared to each other the region which exhibited the largest degree of genetic variation was the middle section of *cps*; the presence of three putative ORFs in this region in ST258 strains indicates that the sugar content in the capsule of ST11 might be very different from that of ST258. Such a difference may account for the discrepancy in the level of prevalence of these genetically related strains in different parts of the world. Future studies should focus on investigating the underlying mechanisms governing their adaptability to different environmental conditions.

**Table 2.** *cps* loci profile of representative *K. pneumoniae* strains of CG258

Strain	STs	Origin	Genetic lineage	*cps* locus				
				Type	Name	Length (bp)	Number of ORFs (start and end genes)	Reference/Accession numbers
GD4	11	China	Clade 3	KL47	*cps*_GD4_	23 953	19 (*galF* to *ugd*)	CP025951
SH-2	11	China	Clade 2	KL64	*cps*_SH-2_	28 193	23 (*galF* to *ugd*)	PJPE00000000
HS11286	11	China	Clade 1	KL103	*cps*_HS11286_	26 763	21 (*galF* to *ugd*)	NC_016845.1
JM45	11	China	Clade 1	KL125	*cps*_JM45_	23 035	18 (*galF* to *ugd*)	NC_022082.1
XL-1	11	China	Clade 1	KL105	*cps*_XL-1_	27 528	22 (*galF* to *ugd*)	GCA_001939845.1
KKBO-4	258	Italy	–	KL107	*cps*_BO-4_	26 587	20 (*galF* to *wzy*)	[41]/HE866751
ATCC BAA-2146	11	USA	–	KL74	*cps*_ATCC BAA-2146_	36 774	26 (*uge* to *gnd*)	[42]/CP006659.2

### Antimicrobial resistance (AMR) genes in ST11 *K. pneumoniae*

**Fig. 5.**
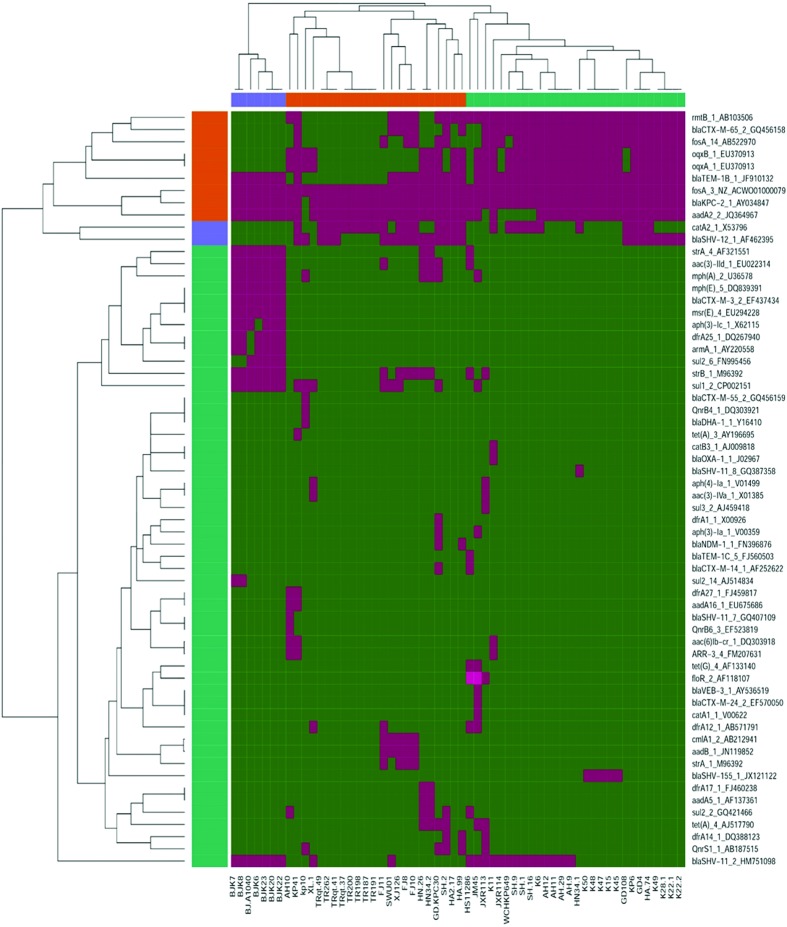
Heatmap of the antimicrobial resistance genes harbored by clinical ST11 *K. pneumoniae* strains from China. The presence of resistance genes in a specific genome is represented by a green box and the absence of resistance genes is represented by a purple box.

**Fig. 6.**
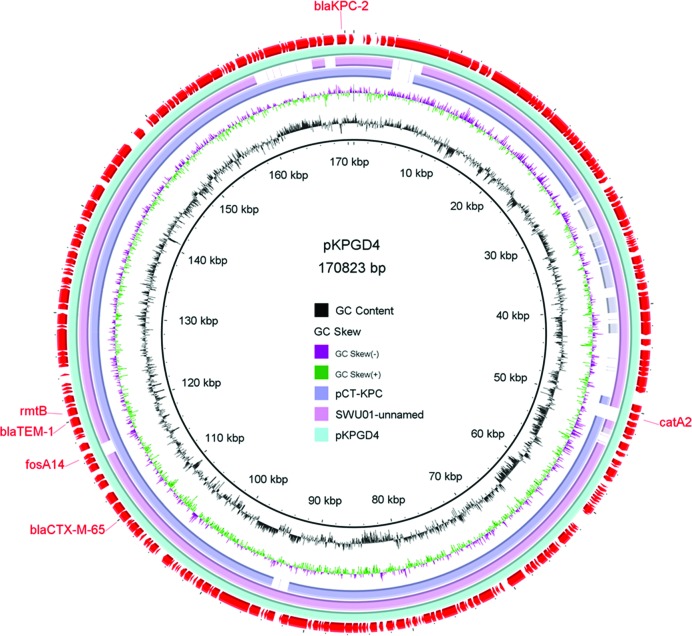
Circular map of plasmid pKPGD4 harbored by *K. pneumoniae* strain GD4. Resistance determinants are indicated in the outermost circle. Plasmids pCT-KPC (accession number: KT185451.1) and SWU01 unnamed plasmid (accession number: CP018455.1) with similar sequence organizations were aligned against pKPGD4.

Dissemination of resistance determinants has been recognized as a major challenge in treatment of bacterial infections worldwide [44]. A targeted survey performed within the 58 ST11 genomes revealed a total of 62 known AMR genes (Fig. 5). The *bla*_SHV_ and *fosA3* genes were shown to be the core chromosomal genes that were present in all the ST11 strains, indicating that these two AMR genes were likely to have been present in the ST11 *K. pneumoniae* ancestor (File S3) [35, 45]. The *oqxA* and *oqxB* genes were both detected in 34 different strains. Being the core genes in *K. pneumoniae*, their role in conferring resistance to fluoroquinolones is not well defined [27, 35]. Other AMR genes that were found to be present in more than 30 strains included *bla*_KPC-2_ (detectable in 57 strains, and only absent in strain kp10), *aadA2* (51), *bla*_TEM-1b_(45), *rmtB* (38), *bla*_CTX-M-65_ (33) and *fosA*-14 (31), conferring resistance to carbapenems, aminoglycosides, β-lactams or fosfomycin, respectively. The test strains were also found to harbor different combinations of the following resistance genes *arr*-3, *qnrB*, *qnrS*, *sul*, *tet*, *str*, *msr*, *mph*, *floR*, *dfrA*, *cat*, *bla*_VEB_, *bla*_TEM_, *bla*_NDM_, *bla*_DHA_, *armA*, *aph* and *aac*, with the number of such genes harbored by each strain ranging from 4 to 17. Undoubtedly, the presence of resistance determinants allows the ST11 strains to survive the barrage of antibiotics used in treatment of hospital infections. The distribution of these genes varied dramatically among strains and was not related to lineage (Fig. 5), indicating that the AMR genes were acquired through horizontal transfer. Due to the constraints of using short-read illumina data [35], we were not able to link each resistance locus to a specific plasmid reliably. However, a search against the PlasmidFinder database [24] allowed us to identify 26 plasmid replicons in the 58 strains (File S5), including 6 colicin replicons and 20 associated with large conjugative AMR plasmids. Importantly, two to seven distinct types of plasmid replicon were detectable in each strain. The *bla*_KPC-2_ genes were found to be frequently associated with the IncFII/IncR plasmid replicons and to have a similar core structure, IS*Kpn27–bla*KPC-2–IS*Kpn6*, as reported in our previous nationwide surveillance study [46]. The completely sequenced plasmid pKPGD4 was found to be an IncFII/IncR-like plasmid, carrying the *bla*_KPC-2_, *bla*_CTX-M-65_, *bla*_TEM-1b_, *rmtB*, *catA2* and *fosA14* genes. The sequence organization of pKPGD4 was similar to that of the unnamed plasmid (accession number: CP018455) in strain *K. pneumoniae* SWU01 (coverage 91 %, identity 99 %) and plasmid pCT-KPC (accession number: KT185451.1, coverage 86 %, identity 99 %) (Fig. 6). This plasmid has been shown to be very common in these ST11 CRKP strains (File S5).

### Prevalence of virulence factors in ST11 *K. pneumoniae*

As an opportunistic pathogen, *K. pneumoniae* relies on an array of virulence factors to colonize and propagate in a host, which include at least (a) surface antigen, especially capsular polysaccharide (CPS, K antigen); (b) siderophores that are responsible for binding ferric iron secreted by the iron-binding proteins of the host; and (c) adherence factors that are responsible for attachment to host cell surfaces, such as type 1 and type 3 fimbriae, and non-fimbrial adhesion proteins [14]. the results of virulence gene analysis of these ST11 strains indicated that some of the virulence genes were commonly present in ST11 strains. The *FimA*, *B*, *C*, *D*, *E*, *F*, *G*, *H*, *I* and *K* genes encoding type 1 fimbriae, which have been reported to be involved in enhancing bacterial virulence during urinary tract infection, were detected in almost all the ST11 strains and ST258 strains. The *mrkA*, *B*, *C*, *D*, *F*, *J*, *H* and *I* genes encoding type 3 fimbriae, which have been shown to mediate biofilm formation on both abiotic and biotic surfaces, as well as *kpn* (coding for FimH-like adhesins) and *ycfM* [coding for outer membrane lipoproteins (OMLs)], both of which have been reported to be involved in bacterial adhesion processes, were also detected in almost all the ST11 strains and ST258 strains [47]. Expression of these genes might therefore enhance the adhesive capacity of *K. pneumoniae* towards respiratory epithelial cells and surfaces of medical devices like ventilators, thus enhancing their ability to cause ventilator-associated infections. Besides, findings of two previous studies revealed a strong relationship between antibiotic resistance and the prevalence of biofilms and OMLs in bacteria, as biofilms and OMLs are known to actively protect bacteria from drug exposure [48, 49]. Carriage of all these virulence determinants might be associated with the high level antimicrobial resistance phenotype of ST11 *K. pneumoniae*. Another outer membrane lipoprotein TraT, encoded by the plasmid-borne transfer gene *traT*, has been previously demonstrated to be able to mediate resistance to bacterial killing by serum. The *traT* gene was found to be frequently associated with production of K1 capsule [50]. Interestingly, the results of virulence factor analysis indicated that some of the ST11 strains also harbored this gene.

**Fig. 7.**
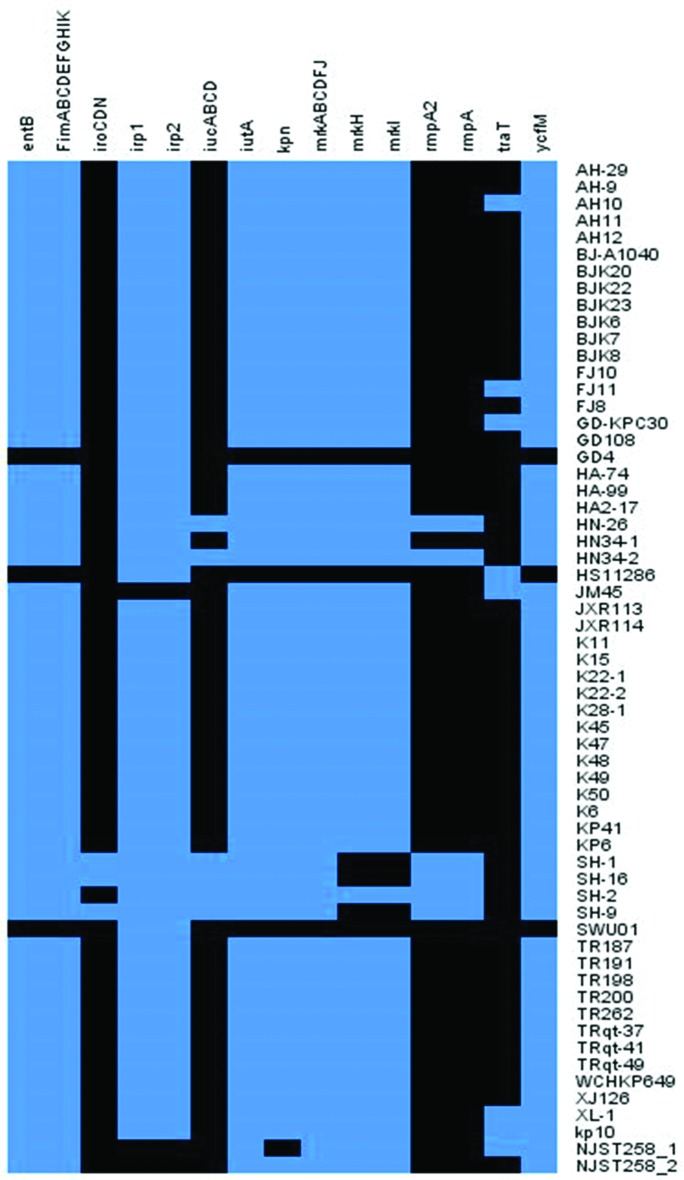
Heatmap of virulence determinants harbored by clinical ST11 *K. pneumoniae* strains from China. The presence of virulence genes in a specific genome is represented by the blue box and the absence of virulence genes is represented by a black box.

Several types of siderophores are known to be differently expressed in ST11 *K. pneumoniae*, with genes encoding enterobactin synthesis, such as *entB*, being present in the majority of ST11 strains [2]. Genes encoding yersiniabactin (the *irp* genes) and its transporters (the *ybt* and *fyu* genes) were detected in majority of the ST11 *K. pneumoniae* genomes in this work, with only one exception (strain JM45, Fig. 7, File S4). Interestingly, these genes were not detectable in the ST258 type of *K. pneumoniae*, constituting a major difference between these two types of strains. The *ybt* locus has been reported to be located in a self-transmissible ICE in the *K. pneumoniae* genome and was significantly associated with infections [27, 35]. Carriage of yersiniabactin-encoding genes in the ST11 strains may pose a significant threat to public health since multidrug-resistant and virulent *K. pneumoniae* strains are causing an increasing number of fatal hospital infections in China. Surprisingly, genes encoding salmochelin (*iroBCDN*), aerobactin (*iucABCD*) and the *rmpA* gene were also detected in a few ST11 genomes. Notably, three strains (HN-26, HN34-2 and SH-2) in phylogenetic clade 2, all of the capsular locus type KL64, were found to harbor the *iucABCD* and *rmpA* genes, and three strains (SH-16, SH-1 and SH-9) in clade 3 of the capsular locus type KL47 were found to contain the gene cluster *iroCDN* in addition to *iucABCD* and *rmpA* (Fig. 6, File S4). Combined carriage of the *iro* and *iuc* gene clusters, as well as the *rmpA* gene, is frequently, but not always linked to the publicly known *K. pneumoniae* virulence plasmid pLVPK, which is uniquely associated with hvKP strains [2, 35, 51]. These strains may belong to the newly emerged ST11 carbapenem-resistant, hypervirulent *K. pneumonia* (ST11-CR-HvKP) indicating the wide spread of this new superbug in different parts of China [52].

### Conclusions

ST11 *K. pneumoniae* has emerged as the dominant KPC-producing clone in PR China [10]. Comprehensive genomic analysis was performed on representative ST11 strains collected from various locations in China. The genomes of these ST11 strains were found to be highly heterogeneous and could be grouped into three major genetic lineages and five different capsular types through phylogenetic analysis. Multiple resistance and virulence determinants were found in the ST11 genomes; such transposable elements are apparently responsible for rendering these strains a severe threat to human health. These data therefore provide insights into the development of prevention, diagnosis and treatment strategies to combat infections caused by ST11 *K. pneumoniae* strains.

## Data bibliography

1. All Genomes sequenced in this study have been deposited in GenBank with Bioproject numbers PRJNA422332 and PRJNA422171.

## Supplementary Data

45 Supplementary File 2
